# Factors related with sarcopenia and sarcopenic obesity among low- and middle-income settings: the 10/66 DRG study

**DOI:** 10.1038/s41598-020-76575-4

**Published:** 2020-11-24

**Authors:** Christina Daskalopoulou, Yu-Tzu Wu, William Pan, Iago Giné Vázquez, Martin Prince, Matthew Prina, Stefanos Tyrovolas

**Affiliations:** 1 Department of Health Service and Population Research, King’s College London, Institute of Psychiatry, Psychology and Neuroscience, London, SE5 8AF UK; 2 Nicholas School of the Environment, Duke University, Durham, NC 27708 USA; 3 Global Health Institute, Duke University, Durham, NC 27708 USA; 4 Parc Sanitari Sant Joan de Déu, Fundació Sant Joan de Déu, Dr. Antoni Pujades, 42, 08830 Sant Boi de Llobregat, Barcelona Spain; 5 Instituto de Salud Carlos III, Centro de Investigación Biomédica en Red de Salud Mental (CIBERSAM), Monforte de Lemos 3-5. Pabellón 11, 28029 Madrid, Spain; 6 School of Nursing, The Hong Kong Polytechnic University, Hong Kong SAR, China

**Keywords:** Epidemiology, Ageing

## Abstract

Sarcopenia and sarcopenic obesity research in low- and middle- income countries (LMICs) is limited. We investigated sarcopenia and sarcopenic obesity prevalence and sociodemographic, bio-clinical and lifestyle factors in LMICs settings. For the purposes of this study, the 10/66 Dementia Research Group follow-up wave information from individuals aged 65 and over in Cuba, Dominican Republic, Peru, Mexico, Puerto Rico, China, was employed and analysed (n = 8.694). Based on indirect population formulas, we calculated body fat percentage (%BF) and skeletal muscle mass index (SMI). Sarcopenia prevalence ranged from 12.4% (Dominican Republic) to 24.6% (rural Peru); sarcopenic obesity prevalence ranged from 3.0% (rural China) to 10.2% (rural Peru). Odds ratios (OR) with 95% confidence intervals (CI) for sarcopenia were higher for men 2.82 (2.22–3.57) and those with higher %BF 1.08 (1.07–1.09), whereas higher number of assets was associated with a decreased likelihood 0.93 (0.87–1.00). OR of sarcopenic obesity were higher for men 2.17 (1.70–2.76), those reporting moderate alcohol drinking 1.76 (1.21–2.57), and those with increased number of limiting impairments 1.54 (1.11–2.14). We observed heterogeneity in the prevalence of sarcopenia and sarcopenic obesity in the 10/66 settings. We also found a variety of factors to be associated with those. Our results reveal the need for more research among the older population of LMICs.

## Introduction

World population ageing patterns are changing, with older people (i.e. 65 years old and over) now being the fastest growing segment^1^. United Nations estimations showed that in 2010 there were 524 million people in the world aged 65 years old and over, whereas projections indicate that this number will increase to 1.5 billion by 2050 (a threefold increase)^2^. World Health Organization (WHO) estimations show that by that moment, 80% of all older people will reside in low- and middle-income countries (LMICs)^3^. As populations around the globe are rapidly ageing, their health and well-being have become a growing public health concern with wide-reaching economic implications.

Physiological changes such as those in muscle and fat mass are strongly related with ageing, and with a plethora of comorbidities^4^. Muscle loss, when is accompanied with decrements in physical performance and/or muscle strength is characterised as sarcopenia^5^; sarcopenic obesity is defined when sarcopenia coexists with excess fat^4^. Sarcopenia is related among other health outcomes with disability, low quality of life and mortality^6^. Smoking habits, physical inactivity and vascular disease have been suggested as some of its related factors^7^. Additionally, sarcopenic obesity among older adults has been related to an increased risk of non-communicable diseases due to the synergistic effect of low lean/high fat mass in the inflammatory-disease related pathway^8^. Apart from its associations with health status, sarcopenia has also considerable independent effects on healthcare expenses. Sarcopenia’s financial burden both to the individual and the healthcare sector has already been reported^9^. For these reasons, the international classification of diseases ICD-10 just 3 years ago recognised sarcopenia as an independent disease^10^.

Even though the scientific interest of sarcopenia and sarcopenic obesity has increased, there is lack of evidence from cross-national epidemiologic data. Specifically, while few original cohort studies from various settings do exist^11^, studies on the epidemiology of sarcopenia and sarcopenic obesity from LMICs, especially from Latin American settings, are limited^7^ and do not always focus on older people. Tyrovolas et al.^8^ investigated data from two nationally-representative multi-country studies on sarcopenia and sarcopenic obesity which allowed for comparisons among diverge settings. However, that study included only five different settings from LMICs (China, Ghana, India, Mexico and Russia); India reported the highest prevalence of sarcopenia, reaching almost 18%.

In the current study, we aimed to estimate sarcopenia and sarcopenic obesity prevalence and to investigate their associations with sociodemographic, bio-clinical and lifestyle factors in settings from six LMICs (Cuba, Dominican Republic, Peru, Mexico, Puerto Rico and China) part of the 10/66 Dementia Research Group (DRG) population-based cohort study^12^. To our knowledge, this analysis constitutes the first effort of investigating sarcopenia, sarcopenic obesity and related factors in Latin American settings. Considering the fast pace of ageing in the area, this study could potentially offer crucial information missing from LMICs.

## Methods

The 10/66 DRG is a multicentre population-based study of ageing and dementia in LMICs^12^. This survey is among the few large population-based studies that apply standard design and procedures across all survey populations. The main objective of the 10/66 study was the assessment of dementia prevalence; secondary objectives included other adverse health outcomes (i.e. mortality, depression, stroke). Baseline surveys of people 65 years old and over were carried out between 2003 and 2010 in prespecified urban and rural sites and follow-up assessments 3 to 5 years later. Urban sites, which represented the typical predominately lower income or mixed neighbourhoods, were purposively selected and consisted of the following sites: Cuba (Havana and Matanzas), Dominican Republic (Santo Domingo), Peru (Lima), Mexico (Mexico City), Puerto Rico (Bayamon) and China (Xicheng, Beijing). Rural sites referred to areas remote from major population centres with agriculture and related trade as the main local industry and included the following: Peru (Cañete Province), Mexico (Morelos State) and China (Daxing, Beijing Province). Response rate in the baseline wave was excellent ranging from 72 to 98% per site; follow-up rate was also high ranging from 78 to 100%. Project resources, including survey questionnaires are available at http://www.alz.co.uk/1066. Ethical approval was obtained from local ethical committees and the King's College London Research Ethics Committee. A signed informed consent, or witnessed consent in case of illiteracy, or next of kin written agreement in case of incapacity, was obtained from all participants.

For the purposes of this study, we analysed information from the follow up wave only due to lack of crucial data for the calculation of sarcopenia and sarcopenic obesity (i.e., weight) in the baseline survey. All methods were carried out in accordance with relevant guidelines and regulations.

### Socio-demographic and lifestyle characteristics

Socio-demographic characteristics included age, sex, education (none, incomplete primary, completed primary, completed secondary, and completed tertiary), marital status (never, married/cohabiting, widowed, divorced/separated), and wealth. The latter was measured as the number of seven different household assets (i.e. car, television, refrigerator, telephone, mains electricity, mains water, plumbed toilet). We also considered the following lifestyle behaviours: smoking (ever or never smoker), level of physical activity (very or fairly against not very or not at all) and alcohol consumption (no alcohol or heavy consumption: ≥ 15 units per week for women and ≥ 21 units per week for men against moderate consumption: 1–14 units per week for women and 1–21 units per week for men)^13^.

### Anthropometric, physical and clinical factors

Body mass index (BMI) was calculated as weight in kilograms divided by height in meters squared.

Percent body fat (%BF) was calculated using a specific formula which has been validated in many populations and ethnic groups^14^. Skeletal muscle mass (SMM) was calculated as the appendicular skeletal muscle mass (ASM) based on the equation proposed by Lee et al.^15^. ASM was further divided by BMI to create a skeletal muscle mass index (SMI)^16^.

Following the criteria used in previous publications, we defined sarcopenia as having low SMM reflected by lower SMI and a slow gait speed^17^. Low SMM was defined as the lowest quintile of the SMI based on sex-stratified values, and slow gait speed referred to the lowest quintile of walking speed (i.e., assessed as the time in seconds needed to walk 10 m) based on height, age, and sex-stratified values^8^. Country-specific cut-offs were only used to determine low SMI as this indicator is likely to be affected by racial differences in body composition^8^. Sarcopenic obesity referred to the sex-standardised highest quintile of %BF in addition to the presence of sarcopenia.

Physical health status was assessed through self-report of a list of 11 commonly occurring physical impairments, and was grouped into none, one to two, and three or more. The number of the self-reported limiting physical impairments was assessed by the following list: arthritis or rheumatism, persistent cough, breathlessness, difficulty breathing or asthma, high blood pressure, heart trouble or angina, stomach or intestine problems, faints or blackouts, paralysis, weakness or loss of one leg or arm, skin disorders such as pressure sores leg ulcers or severe burns. Dementia was diagnosed by the cross-culturally developed, calibrated and validated 10/66 dementia diagnosis algorithm^12^ and the Diagnostic and Statistical Manual of Mental Disorders (DSM-IV) manual. Depression was assessed with the cross-culturally validated EURO-D scale, derived from the Geriatric Mental State examination^18^. Diabetes and stroke were defined from self-reported medical diagnosis either from the baseline or the follow-up wave.

### Statistical analysis

We calculated sarcopenia and sarcopenic obesity prevalence in each site. We then calculated the characteristics of the study sample by site and by sarcopenic event (i.e. no sarcopenia, sarcopenia, sarcopenic obesity). We used chi-square tests or analysis of variance (categorical variables) to test the differences among the events. We used multivariable linear regressions, where SMI was the outcome, to assess the association between skeletal muscle mass and the following factors: age, education, marital status, number of assets, body fat, physical activity, smoking, drinking and number of physical limiting impairments. Linear regression models were performed per gender as the assumption of normality was not met in the total sample. Our models were also adjusted for the following chronic diseases: dementia, depression, stroke and diabetes. Finally, we performed multivariable logistic regressions where sarcopenia and sarcopenic obesity were considered as the binary outcomes. All analyses were produced per site and then combined with fixed-effects meta-analysis to provide a pooled result. To estimate the proportion of between site variability in estimates accounted for heterogeneity, we computed Higgins I^2^^19^.Values of 50% are considered as moderate heterogeneity, whereas values of 75% are considered as high heterogeneity. The analyses were performed with STATA version 14.1.

Due to high presence of missing data in the variable ‘weight’ in Cuba and Puerto Rico (web-Appendix 1), we imputed this variable by using multiple imputation. This technique replaces missing values with randomly selected draws from the missing data distribution conditional on the observed data. We generated 50 imputed datasets which were analysed separately, and their results were combined. We used ‘mi impute regress’ STATA command, as weight is a continuous variable and we included as auxiliary the following variables: age, height, gender, education level, marital status, number of assets, physical activity, alcohol drinking, current smoking, time taken to walk 10 m, dementia, depression, stroke and diabetes.

## Results

Skeletal muscle mass cut-offs (as reflected by SMI) for women ranged from 0.515 (Mexico) to 0.572 (Cuba), whereas for men ranged from 0.882 (Peru) to 0.964 (Cuba). Cut-offs per site and gender are presented in the web-appendix 2. The highest crude prevalence of sarcopenia was observed in Peru rural site (24.6%, 95%CI 20.4–28.8%); Dominican Republic showed the lowest prevalence (12.4%, 95%CI 10.4–14.3%). Regarding sarcopenic obesity, Peru rural also indicated the highest crude prevalence (10.2%, 95%CI 7.3–13.2%) and China rural site the lowest (3.0%, 95%CI 1.8–4.3%). The prevalence of sarcopenia and sarcopenic obesity per site is presented in Table 1. Table 2 presents the characteristics of the sample by the presence of sarcopenia and sarcopenic obesity. In all sites, those with sarcopenia or sarcopenic obesity had advanced age. In many sites, sarcopenia or sarcopenic obesity event was associated with men, lower education level, widowhood and medium or low individual wealth. Among others, people with sarcopenia or sarcopenic obesity were less physically active in Peru (rural and urban) and rural Mexico and reported a higher number of limiting illnesses in Peru (urban) and China (urban). Those with sarcopenic obesity had higher number of moderate alcohol consumption in Mexico (rural).

**Table 1:** Sarcopenia and sarcopenic obesity prevalence per site.

Sites	Sarcopenia prevalence (and 95% CI)			Sarcopenia obesity prevalence (and 95% CI)		
Cuba	12.9%	11.3%	14.4%	5.1%	4.0%	6.2%
Dominican Republic	12.4%	10.4%	14.3%	5.2%	3.8%	6.5%
Peru (urban)	14.6%	12.2%	17.1%	6.5%	4.8%	8.2%
Peru (rural)	24.6%	20.4%	28.8%	10.2%	7.3%	13.2%
Mexico (urban)	14.9%	12.2%	17.6%	6.0%	4.2%	7.8%
Mexico (rural)	15.9%	13.1%	18.8%	4.1%	2.6%	5.6%
China (urban)	18.9%	16.0%	21.9%	8.2%	6.2%	10.3%
China (rural)	15.0%	12.4%	17.7%	3.0%	1.8%	4.3%
Puerto Rico	16.7%	14.3%	19.1%	5.2%	3.8%	6.7%

**Table 2:** Characteristics of the study sample.

Cross-sectional characteristics		Cuba				Dominican Republic				Peru (urban)				Peru (rural)				Mexico (urban)			
Characteristic	Category	Number of obs	No sarcopenia	Sarcopenia only	Sarcopenic obesity	Number of obs	No sarcopenia	Sarcopenia only	Sarcopenic obesity	Number of obs	No sarcopenia	Sarcopenia only	Sarcopenic obesity	Number of obs	No sarcopenia	Sarcopenia only	Sarcopenic obesity	Number of obs	No sarcopenia	Sarcopenia only	Sarcopenic obesity
Age	65–69	**592**	**34.2%**	**9.8%**	**8.3%**	**343**	**34.1%**	**12.8%**	**16.1%**	**241**	**33.4%**	**2.9%**	**3.7%**	**147**	**44.0%**	**11.9%**	**9.5%**	**190**	**31.4%**	**4.9%**	**9.8%**
	70–74	**562**	**31.3%**	**18.3%**	**14.6%**	**332**	**31.7%**	**16.7%**	**32.1%**	**230**	**29.6%**	**14.7%**	**18.5%**	**111**	**28.8%**	**20.3%**	**23.8%**	**242**	**37.3%**	**26.2%**	**22.0%**
	75–79	**414**	**21.3%**	**23.5%**	**25.0%**	**212**	**18.9%**	**26.9%**	**19.6%**	**186**	**23.1%**	**13.2%**	**24.1%**	**76**	**16.2%**	**18.6%**	**35.7%**	**137**	**18.4%**	**26.2%**	**34.1%**
	80 +	**345**	**13.3%**	**48.4%**	**52.1%**	**198**	**15.4%**	**43.6%**	**32.1%**	**175**	**13.9%**	**69.1%**	**53.7%**	**76**	**11.0%**	**49.2%**	**31.0%**	**115**	**12.9%**	**42.6%**	**34.1%**
Gender	Female	**1261**	**65.2%**	**75.2%**	**60.4%**	**746**	**69.5%**	**76.9%**	**44.6%**	546	66.0%	70.6%	53.7%	221	51.5%	64.4%	57.1%	**451**	**67.2%**	**67.2%**	**46.3%**
	Male	**657**	**34.8%**	**24.8%**	**39.6%**	**339**	**30.5%**	**23.1%**	**55.4%**	287	34.0%	29.4%	46.3%	189	48.5%	35.6%	42.9%	**233**	**32.8%**	**32.8%**	**53.7%**
Education	None	**39**	**1.9%**	**3.3%**	**3.2%**	207	18.4%	24.4%	25.0%	**19**	**1.6%**	**5.9%**	**7.4%**	**59**	**11.5%**	**29.3%**	**16.7%**	**132**	**17.8%**	**32.8%**	**22.0%**
	Some, did not complete primary	**390**	**18.8%**	**30.1%**	**31.6%**	545	50.2%	57.7%	44.6%	**56**	**5.7%**	**13.2%**	**13.0%**	**101**	**24.0%**	**25.9%**	**31.0%**	**258**	**36.0%**	**52.5%**	**41.5%**
	Completed primary	**622**	**32.0%**	**35.3%**	**35.8%**	206	19.5%	14.1%	19.6%	**261**	**30.6%**	**39.7%**	**33.3%**	**203**	**53.3%**	**36.2%**	**47.6%**	**157**	**24.7%**	**9.8%**	**19.5%**
	Completed secondary	**525**	**28.5%**	**24.2%**	**12.6%**	78	7.6%	2.6%	7.1%	**304**	**38.8%**	**20.6%**	**29.6%**	**29**	**8.2%**	**5.2%**	**2.4%**	**73**	**11.2%**	**4.9%**	**12.2%**
	Tertiary (college)	**340**	**18.8%**	**7.2%**	**16.8%**	44	4.3%	1.3%	3.6%	**189**	**23.5%**	**20.6%**	**16.7%**	**12**	**3.0%**	**3.4%**	**2.4%**	**62**	**10.3%**	**0.0%**	**4.9%**
marital status	Never married	**174**	**8.9%**	**9.8%**	**11.6%**	75	6.8%	5.1%	12.5%	89	10.6%	14.9%	7.4%	**50**	**12.0%**	**13.6%**	**11.9%**	42	5.8%	9.8%	4.9%
	Married/cohabiting	**867**	**46.6%**	**33.3%**	**41.1%**	350	32.4%	24.4%	42.9%	487	59.6%	56.7%	53.7%	**238**	**62.3%**	**39.0%**	**54.8%**	344	52.2%	31.1%	51.2%
	Widowed	**560**	**27.3%**	**45.8%**	**36.8%**	423	39.1%	50.0%	23.2%	213	25.1%	26.9%	33.3%	**106**	**22.4%**	**39.0%**	**33.3%**	245	34.2%	50.8%	36.6%
	Divorced/separated	**315**	**17.3%**	**11.1%**	**10.5%**	234	21.7%	20.5%	21.4%	37	4.7%	1.5%	5.6%	**15**	**3.2%**	**8.5%**	**0.0%**	53	7.7%	8.2%	7.3%
ASSETS	0, 1, 2	13	0.7%	0.7%	0.0%	69	6.6%	6.4%	1.8%	1	0.1%	0.0%	0.0%	**16**	**2.3%**	**11.9%**	**4.8%**	6	**0.7%**	**0.0%**	**4.9%**
	3, 4, 5	467	24.5%	27.5%	17.7%	474	44.3%	42.3%	35.7%	17	1.7%	4.4%	3.7%	**200**	**46.9%**	**47.5%**	**64.3%**	91	**11.7%**	**29.5%**	**12.2%**
	6, 7	1432	74.7%	71.9%	82.3%	542	49.1%	51.3%	62.5%	815	98.2%	95.6%	96.3%	**194**	**50.8%**	**40.7%**	**31.0%**	587	**87.6%**	**70.5%**	**82.9%**
Physical activity	Not very/not at all	647	33.5%	40.0%	42.4%	374	35.3%	35.1%	29.1%	**317**	**35.4%**	**57.4%**	**48.1%**	**114**	**21.0%**	**47.5%**	**50.0%**	251	35.8%	41.7%	43.9%
	Very/failry	1233	66.5%	60.0%	57.6%	697	64.7%	64.9%	70.9%	**516**	**64.6%**	**42.6%**	**51.9%**	**296**	**79.0%**	**52.5%**	**50.0%**	431	64.2%	58.3%	56.1%
Smoking	Never	1668	87.3%	89.5%	92.7%	971	89.7%	92.3%	91.1%	799	95.6%	100.0%	94.4%	404	98.1%	100.0%	100.0%	622	90.9%	95.1%	85.4%
	Ever	233	12.7%	10.5%	7.3%	108	10.3%	7.7%	8.9%	34	4.4%	0.0%	5.6%	6	1.9%	0.0%	0.0%	62	9.1%	4.9%	14.6%
Alcohol Group	No drinking/heavy	1824	96.5%	97.3%	93.8%	1018	96.0%	96.2%	89.3%	774	92.5%	97.1%	94.4%	405	98.7%	98.3%	100.0%	605	88.6%	90.2%	85.4%
	Moderate drinking	37	3.5%	2.7%	6.3%	46	4.0%	3.8%	10.7%	58	7.5%	2.9%	5.6%	5	1.3%	1.7%	0.0%	78	11.4%	9.8%	14.6%
Number of limiting illnesses	0	819	43.1%	42.5%	40.6%	386	36.9%	29.5%	23.2%	**455**	**56.1%**	**48.5%**	**42.6%**	268	68.3%	54.2%	59.5%	279	41.4%	42.6%	29.3%
	One-two	870	45.7%	47.7%	40.6%	480	43.5%	47.4%	53.6%	**293**	**34.9%**	**38.2%**	**35.2%**	122	27.2%	39.0%	35.7%	292	41.4%	50.8%	48.8%
	More than two	220	11.3%	9.8%	18.8%	217	19.6%	23.1%	23.2%	**85**	**9.0%**	**13.2%**	**22.2%**	20	4.5%	6.8%	4.8%	113	17.2%	6.6%	22.0%

Cross-sectional characteristics		Mexico (rural)				China (urban)				China (rural)				Puerto Rico							
Characteristic	Category	Number of obs	No sarcopenia (%)	Sarcopenia only (%)	Sarcopenic obesity (%)	Number of obs	No sarcopenia (%)	Sarcopenia only (%)	Sarcopenic obesity (%)	Number of obs	No sarcopenia (%)	Sarcopenia only (%)	Sarcopenic obesity (%)	Number of obs	No sarcopenia (%)	Sarcopenia only (%)	Sarcopenic obesity (%)				
Age	65–69	**209**	**36.6**	**10.7**	**23.1**	**235**	**38.9**	**13.5**	**12.3**	**316**	**49.3**	**24.1**	**28.6**	**222**	**27.6**	**5.8**	**15.2**				
	70–74	**175**	**28.3**	**20.0**	**34.6**	**230**	**35.8**	**18.9**	**26.3**	**213**	**31.0**	**31.3**	**23.8**	**255**	**30.3**	**18.3**	**15.2**				
	75–79	**132**	**19.5**	**33.3**	**11.5**	**150**	**18.0**	**43.2**	**29.8**	**119**	**15.0**	**31.3**	**23.8**	**246**	**27.0**	**26.0**	**32.6**				
	80+	**118**	**15.6**	**36.0**	**30.8**	**77**	**7.3**	**24.3**	**31.6**	**44**	**4.8**	**13.3**	**23.8**	**184**	**15.2**	**50.0**	**37.0**				
Gender	Female	**391**	**61.7**	**70.7**	**34.6**	**410**	**60.8**	**67.6**	**33.3**	394	57.1	59.0	42.9	**626**	**68.8**	**77.9**	**56.5**				
	Male	**243**	**38.3**	**29.3**	**65.4**	**282**	**39.2**	**32.4**	**66.7**	298	42.9	41.0	57.1	**278**	**31.2**	**22.1**	**43.5**				
Education	None	210	32.1	40.0	34.6	140	19.6	27.0	17.5	376	53.2	60.2	61.9	19	1.9	4.8	0.0				
	Some, did not complete primary	319	51.0	46.7	46.2	98	14.1	17.6	10.5	85	12.1	14.5	9.5	154	16.7	22.1	10.9				
	Completed primary	74	11.3	12.0	19.2	168	23.2	27.0	31.6	191	28.6	22.9	19.0	164	17.5	23.1	17.4				
	Completed secondary	21	3.8	1.3	0.0	211	32.3	18.9	28.1	37	5.6	2.4	9.5	364	41.4	31.7	41.3				
	Tertiary (college)	10	1.9	0.0	0.0	75	10.9	9.5	12.3	3	0.5	0.0	0.0	203	22.6	18.3	30.4				
marital status	Never married	17	2.8	2.7	0.0	**1**	**0.2**	**0.0**	**0.0**	19	2.4	4.8	4.8	**58**	**6.8**	**4.8**	**4.4**				
	Married/cohabiting	359	57.7	48.0	61.5	**519**	**77.7**	**59.5**	**68.4**	431	64.6	48.2	52.4	**479**	**54.6**	**36.5**	**63.0**				
	Widowed	225	35.2	38.7	34.6	**172**	**22.1**	**40.5**	**31.6**	241	32.8	47.0	42.9	**254**	**26.0**	**47.1**	**19.6**				
	Divorced/separated	32	4.3	10.7	3.8					1	0.2	0.0	0.0	**113**	**12.6**	**11.5**	**13.0**				
ASSETS	0, 1, 2	**103**	**14.1**	**29.3**	**23.1**	4	0.5	1.4	0.0	**8**	**0.7**	**2.4**	**9.5**	10	1.2	1.0	0.0				
	3, 4, 5	**364**	**58.3**	**48.0**	**65.4**	268	38.5	43.2	35.1	**231**	**33.0**	**38.6**	**23.8**	10	1.1	1.9	0.0				
	6, 7	**167**	**27.6**	**22.7**	**11.5**	420	61.0	55.4	64.9	**453**	**66.3**	**59.0**	**66.7**	885	97.8	97.1	100.0				
Physical activity	Not very/not at all	**299**	**45.4**	**50.7**	**73.1**	348	48.5	58.1	57.9	352	49.5	61.4	47.6	399	43.0	49.0	55.6				
	Very/fairly	**335**	**54.6**	**49.3**	**26.9**	344	51.5	41.9	42.1	340	50.5	38.6	52.4	501	57.0	51.0	44.4				
Smoking	Never	609	96.2	96.0	92.3	573	82.5	81.1	87.7	483	68.8	76.5	81.0	878	97.1	99.0	100.0				
	Ever	25	3.8	4.0	7.7	119	17.5	18.9	12.3	206	31.2	23.5	19.0	23	2.9	1.0	0.0				
Alcohol Group	No drinking/heavy	**595**	**93.8**	**98.7**	**84.6**	654	94.5	98.6	89.5	591	85.8	89.2	66.7	823	91.8	92.3	88.9				
	Moderate drinking	**38**	**6.2**	**1.3**	**15.4**	38	5.5	1.4	10.5	99	14.2	10.8	33.3	74	8.2	7.7	11.1				
Number of limiting illnesses	0	209	33.0	32.0	34.6	**199**	**30.5**	**17.6**	**26.3**	330	48.3	45.8	38.1	233	25.6	26.9	26.1				
	One-two	298	46.7	50.7	42.3	**314**	**46.5**	**45.9**	**33.3**	299	42.2	50.6	42.9	454	51.8	43.3	41.3				
	More than two	127	20.3	17.3	23.1	**179**	**23.0**	**36.5**	**40.4**	63	9.5	3.6	19.0	216	22.6	29.8	32.6				

Bold cells indicate a statistically significant result at alpha = 0.05.

Table 3 presents the association between SMI (ASM/BMI), which reflects SMM, and a variety of factors. In the pooled results, higher levels of education and wealth were related with higher SMI, while being older and higher %BF was associated with lower SMI (i.e., lower SMM), both in men and women. Higher %BF was consistently associated with lower SMI in all areas in men and women, except China (in women only) and Cuba (in men only) (web-appendix-Table 3 (per site)). All other factors pictured regional variability in their association with SMI as this was also reflected by the considerable heterogeneity (I^2^). Heterogeneity in the meta-analytical results ranged from low (0.0%) to high (91.8%) in women, and from low (0.0%) to high (60.8%) in men.

**Table 3:** Skeletal muscle associations estimated by multivariable linear regression model.

Characteristic	Females		Males	
	Meta-analysed coefficients (95% CI)	I^2^ (p-value)	Meta-analysed coefficients (95% CI)	I^2^ (p-value)
Age	− 0.005 (− 0.006, − 0.005)	31.8% (0.164)	-0.003 (− 0.003, − 0.002)	8.4% (0.365)
**Education (none)**	Reference			
Some, did not complete primary	0.003 (− 0.004, 0.010)	17.2% (0.289)	0.000 (− 0.012, 0.011)	29.4% (0.183)
Completed primary	0.014 (0.007, 0.022)	0.0% (0.539)	0.005 (− 0.007, 0.016)	19.9% (0.266)
Completed secondary	0.025 (0.015, 0.035)	53.6% (0.028)	0.021 (0.007, 0.035)	33.6% (0.149)
Tertiary (college)	0.064 (0.053, 0.074)	91.8% (< 0.001)	0.038 (0.023, 0.054)	55.4% (0.022)
**Marital status (never married)**	Reference			
Married/ cohabiting	0.005 (− 0.003, 0.014)	79.6% (< 0.001)	0.004 (− 0.010, 0.019)	0.0% (0.528)
Widowed	0.006 (− 0.003, 0.015)	78.5% (< 0.001)	0.004 (− 0.011, 0.028)	0.0% (0.457)
Divorced/ separated	0.010 (0.000, 0.021)	67.8% (0.005)	0.007 (− 0.012, 0.025)	37.5% (0.163)
**Number of assets**	0.003 (0.001, 0.005)	57.3% (0.016)	0.005 (0.002, 0.008)	0.0% (0.502)
Body fat	− 0.003 (− 0.003, − 0.002)	88.3% (< 0.001)	− 0.013 (− 0.014, − 0.013)	19.3% (0.222)
**Physical activity (not at all/not very)**	Reference			
Fairly/very	0.004 (− 0.001, 0.009)	45.1% (0.068)	0.002 (− 0.005, 0.010)	0.0% (0.485)
**Drinking (no drinking/heavy)**	Reference			
Moderate drinking	0.041 (− 0.006, 0.014)	0.0% (0.528)	− 0.003 (− 0.013, 0.006)	34.9% (0.139)
**Smoking (never)**	Reference			
Ever	0.005 (− 0.003, 0.014)	0.0% (0.492)	0.005 (− 0.004, 0.014)	0.0% (0.721)
**Number of limiting impairments (none)**	Reference			
One-two	− 0.004 (− 0.008, 0.001)	28.9% (0.188)	0.001 (− 0.006, 0.009)	55.6% (0.021)
More than two	0.001 (− 0.006, 0.007)	55.6% (0.021)	0.001 (− 0.010, 0.012)	60.8% (0.009)

Models were adjusted for dementia, depression, diabetes and stroke.

The association between sarcopenia and various factors, estimated by multivariable logistic regression, is presented in Table 4. The meta-analysed findings indicated that higher number of assets (OR: 0.93, 95%CI 0.87–1.00) and being married or widowed (OR: 0.68, 95%CI 0.51–0.92; OR: 0.74, 95%CI 0.56–0.96, respectively) were associated with lower odds of sarcopenia. On the contrary, higher %BF (OR:1.08, 95%CI 1.07–1.09), older age (OR: 1.14, 95%CI 1.12–1.15) and men (OR: 2.82, 95%CI 2.22–3.57) were associated with increased odds of sarcopenia. Heterogeneity among sites ranged from low (0.0%) to moderate (58.8%). Table 4 also presents the associations between sarcopenic obesity and various factors, estimated by multivariable logistic regression. The pooled results showed that older age (OR:1.11, 95%CI 1.09–1.13), men (OR: 2.17, 95%CI 1.70–2.76), moderate alcohol drinking (OR: 1.76, 95%CI 1.21–2.57) and higher number of limiting impairments (OR: 1.54, 95%CI 1.11–2.14) were significantly associated with higher odds of sarcopenic obesity. Heterogeneity among sites ranged from low (0.0%) to moderate (52.0%).

**Table 4:** Aggregated associations of sarcopenia and sarcopenic obesity.

	Sarcopenia		Sarcopenic Obesity	
	Pooled estimates for odds ratios (95% CI)	I^2^ (p-value)	Pooled estimates for odds ratios (95% CI)	I^2^ (p-value)
Age	1.14 (1.12, 1.15)	6.5% (0.382)	1.11 (1.09, 1.13)	37.5% (0.097)
**Gender (females)**	Reference			
Males	2.82 (2.22, 3.57)	22.7% (0.234)	2.17 (1.70, 2.76)	36.4% (0.127)
**Education (none)**	Reference			
Some, did not complete primary	0.96 (0.75, 1.24)	0.0% (0.975)	0.81 (0.55, 1.17)	0.0% (0.735)
Completed primary	0.87 (0.67, 1.14)	3.8% (0.403)	0.92 (0.63, 1.33)	0.2% (0.432)
Completed secondary	0.68 (0.47, 0.97)	0.0% (0.901)	0.97 (0.60, 1.56)	4.1% (0.398)
Tertiary (college)	0.60 (0.37, 0.96)	0.0% (0.769)	0.66 (0.35, 1.22)	0.0% (0.843)
**Marital status (never married)**	Reference			
Married/cohabiting	0.68 (0.51, 0.92)	0.0% (0.663)	0.90 (0.62, 1.30)	0.0% (0.969)
Widowed	0.74 (0.56, 0.96)	0.0% (0.781)	0.82 (0.52, 1.30)	0.0% (0.449)
Divorced/ separated	0.99 (0.66, 1.46)	0.0% (0.541)	1.17 (0.76, 1.80)	0.0% (0.615)
**Number of assets**	0.93 (0.87, 1.00)	53.1% (0.029)	1.03 (0.93, 1.15)	41.9% (0.088)
Body fat (%BF)	1.08 (1.07, 1.09)	58.8% (0.013)	–	
**Physical activity (not at all/not very)**	Reference			
Fairly/very	1.01 (0.86, 1.19)	58.1% (0.014)	0.93 (0.72, 1.21)	52.0% (0.042)
**No drinking/heavy**	Reference			
Moderate drinking	1.21 (0.91, 1.62)	17.4% (0.288)	1.76 (1.21, 2.57)	29.8% (0.190)
**Smoking (never)**	Reference			
Ever	0.95 (0.72, 1.25)	0.0% (0.624)	0.83 (0.59, 1.18)	25.5% (0.225)
**Number of limiting impairments (none)**	Reference			
One-two	0.99 (0.84, 1.17)	5.5% (0.326)	1.00 (0.77, 1.29)	0.0% (0.554)
More than two	1.02 (0.81, 1.29)	27.4% (0.112)	1.54 (1.11, 2.14)	0.0% (0.478)

Models were adjusted for dementia, depression, diabetes and stroke.

## Discussion

The current work revealed variability in the prevalence of sarcopenia and sarcopenic obesity among adults aged ≥ 65 years, living in LMICs. Higher %BF was related with sarcopenia and lower SMM in the majority of sites. In addition, our pooled results showed that marital status (married/cohabiting and widowed) and wealth level (estimated by number of assets) were significantly associated with lower odds for sarcopenia. Men also showed an increased likelihood of sarcopenia event. We also observed an association of sarcopenic obesity with the number of limiting impairment disorders and moderate drinking. The current work also revealed common sarcopenia and sarcopenic obesity risk factor patterns between LMICs of Latin America and other regions (i.e., Asia, Africa)^8^. However, the association between marital status and sarcopenia as well as of moderate alcohol drinking and sarcopenic obesity are reported for the first time among LMICs picturing a regional diversity that international health policy makers should take into account.

To the best of our knowledge, this study is among the first cross-national studies that estimated sarcopenia and sarcopenic obesity prevalence in LMICs and the first one reporting estimates for Peru, Cuba, Dominican Republic, and Puerto Rico. The prevalence of sarcopenia varied from 24.6% (rural Peru) to 12.4% (Dominican Republic) while the prevalence of sarcopenic obesity was 3.0% (rural China) to 10.2% (rural Peru). These figures are in agreement with previously reported data in different areas^8^. Other studies reported sarcopenia prevalence between 5 and 50% depending on sample’s age^20^, and sarcopenic obesity prevalence between 3 and 12%^21^. In their previous study Tyrovolas et al. found sarcopenia prevalence from 12.9% (Poland) to almost 17.5% (India) and sarcopenic obesity from 1.3% (India) to 11.0% (Spain)^8^.

We also investigated the associations of these events and of skeletal muscle mass with a variety of factors (clinical, anthropometric, socio-demographic, and lifestyle) using a large dataset of older adults from the aforementioned settings. This allowed for a direct comparison of different LMICs which has not been done previously. The predictors of low SMM and sarcopenia shared a common nature. This may be due to the mutual physiological pathway where low SMM later on could lead to sarcopenia^22^. Sarcopenia and SMM, were consistently related with %BF and marital status, education and/or wealth. Marital status pictured a diverse association with SMM in by-site analysis, but it was protective against sarcopenia in the pooled estimates. This has not been reported previously and constitutes an area for further research. This relation could follow the same effect on individual´s health and probability of death, as described by numerous studies for marital and living status, and its changes during mid-life, especially after the age of 65 in LMICs^23^. Although the association between marital status and sarcopenia is reported for the first time, previous studies have suggested an association between sarcopenia or low lean mass with lower socio-economic level^7^.

We did not find a clear effect of physical activity on SMM and there was no association with sarcopenia in the pooled analysis. Research has indicated that specialised intervention programs (i.e. resistance exercise) are able to manage, or even slow down, sarcopenia and its negative health outcomes^24^, mostly through improvements in muscle oxygenation. Previous studies in the field have also reported a relationship between physical activity and sarcopenia^8^, however some others found no association^25^. Since the definition of sarcopenia in this research work includes only the component of gait speed in addition to muscle mass, it may have been that those with low functionality were not able to perform physical exercise at higher levels^26^. Future studies are needed to assess whether these results could be replicated.

Men had consistently higher odds than women for sarcopenia and sarcopenic obesity with the strongest association in urban Peru. This gender and sarcopenia/sarcopenic obesity association could be partly explained by the different nature of ageing (i.e., endocrine, muscle tissue) among genders. It is well known that functional capacity (that could be reflected through gait speed) has gender differences with females having better levels of functionality^27^. In addition, we also found that higher BF% was related with both low SMM and sarcopenia. This factor is consistent among High- and LMICs and could be explained by the clinical pathway of low-grade inflammation by excess body fat tissue, low lean mass, and consequently of sarcopenia^8^.

Moderate alcohol drinking and the accumulated number of impairments were the only potentially modifiable factors that were significantly associated with sarcopenic obesity. While the meta-analysed estimate of sarcopenia was not related with number of impairments, the pooled estimate of sarcopenic obesity was strongly associated with them. Sarcopenic obesity combines high fat with low skeletal mass and is related with a variety of metabolic, endocrine and lifestyle factors^4^. However, while alcohol intake and obesity have been extensively studied, whether alcohol drinking is a potential risk factor for sarcopenia is still unclear^28^. It has been reported that ethanol inhibits protein synthesis in muscles while mutually increases muscle autophagy^29^. A well conducted study recently reported the relation between alcohol drinking and cachexia^30^, although actual cross-national data on sarcopenia and alcohol are scarce.

Sarcopenia and sarcopenic obesity are health concepts that could be reversed with specific lifestyle intervention planning^8,24^. Apart from lifestyle changes, early prevention and well targeted intervention strategies at middle life, focusing on specific disorders (i.e., arthritis, hypertension, respiratory infections) could also be effective against sarcopenic obesity. Older people especially in low income areas are facing challenges that affect their social and economic well-being and consequently their health. Yet, this population group often is not prioritised by health programs and policies^31^. The scenery in LMICs is even more complex, if we consider the pace of population ageing and the metabolic related disease rise, along with limited healthcare services access. As a result, early preventive lifestyle measures (e.g., alcohol drinking) aiming to promote healthy ageing and minimise the risk of sarcopenia and sarcopenic obesity are required.

## Strengths and limitations

To the best of our knowledge, this is the first study that evaluated the effect of various factors with SMM, sarcopenia, and sarcopenic obesity, by using large samples of older people residing in Latin American settings and China (taking into account rural and urban settings). Nevertheless, our findings should be interpreted within the limitations inherited with our dataset. Firstly, the fact that this is a cross-sectional analysis limits the potential investigation of etiological mechanisms. Secondly, we estimated prevalence by using data from the follow-up wave as crucial information to calculate sarcopenia was not available in the baseline survey. In addition, 10/66 survey included only selected catchment urban and rural sites of the countries under investigation. Therefore, these estimates may not be generalisable across the whole country even though 10/66 assessors had undertaken substantial training to ensure consistency across survey sites. Also, estimates of %BF and ASM were based on all ages population equations which slightly differ from the current study’s population sample (for instance in mean age), and not in a direct assessment. Yet, these equations have reported good concordance rates when compared with gold standard methods. In addition to that, indirect assessments of muscle mass are the only cost-effective way among population-based studies in comparison with other classical techniques. For the evaluation of sarcopenia, we used walking speed without the use of grip strength (as also has been suggested), due to lack of this measurement in the 10/66 surveys. Another limitation of our study is that our data did not allow the calculation of sarcopenia by employing biochemical markers as Yang et al.^32^ have applied in previous publications^32^. Finally, even though nutrition and specific macro-nutrients consumption, such as protein intake, are associated with muscle mass, the 10/66 survey did not include a comprehensive dietary assessment allowing the extraction of macronutrients for further analysis.

## Conclusions

In this study, we examined the role of various risk factors on skeletal muscle mass, sarcopenia, and sarcopenic obesity among older populations residing in LMICs. As it is expected that in 2050 80% of the older population will live in less developed areas of the world, it is crucial to investigate the role of sarcopenia and sarcopenic obesity as a part of the ageing process. Our findings suggested that there may be significant associations of gender, marital status, education, wealth and body fat with changes in SMM and consequently in the likelihood of sarcopenia. Moreover, the fact that accumulated limiting impairments and alcohol drinking were related with sarcopenic obesity points directly to targeted health prevention strategies and lifestyle intervention policies. More research is needed to understand the moderating pathway of socio-demographic factors on sarcopenia in low- and middle- income ageing populations and especially among Latin American settings. As LMICs have limited healthcare and primary prevention resources, sustainable and well-targeted health promotion programs (e.g., low alcohol intake promotion, central obesity prevention, physical activity) may constitute cost-effective means for the promotion of low- or free- of sarcopenia ageing context.

## Supplementary information

Supplementary Information.

## Data Availability

The 10/66 Dementia Research Group dataset is available upon request via the official site of the study: https://www.alz.co.uk/1066. All data generated in this study are available from the corresponding author on request.
